# Mechanism of contraction rhythm homeostasis for hyperthermal sarcomeric oscillations of neonatal cardiomyocytes

**DOI:** 10.1038/s41598-020-77443-x

**Published:** 2020-11-24

**Authors:** Seine A. Shintani, Takumi Washio, Hideo Higuchi

**Affiliations:** 1 Department of Biomedical Sciences, College of Life and Health Sciences, Chubu University, 1200 Matsumoto-cho Kasugai, Aichi, 487-8501 Japan; 2 UT-Heart Inc., 178-4-4 Wakashiba, Kashiwa, 277-0871 Japan; 3 Future Center Initiative, The University of Tokyo, 178-4-4 Wakashiba, Kashiwa, 277-0871 Japan; 4 Department of Physics, Graduate School of Science, The University of Tokyo, 7-3-1 Hongo Bunkyo-ku, Tokyo, 113-0033 Japan

**Keywords:** Biophysics, Computational biophysics, Molecular biophysics, Motility, Nanoscale biophysics, Cardiovascular biology, Circulation, Cellular imaging

## Abstract

The heart rhythm is maintained by oscillatory changes in [Ca^2+^]. However, it has been suggested that the rapid drop in blood pressure that occurs with a slow decrease in [Ca^2+^] preceding early diastolic filling is related to the mechanism of rapid sarcomere lengthening associated with spontaneous tension oscillation at constant intermediate [Ca^2+^]. Here, we analyzed a new type of oscillation called hyperthermal sarcomeric oscillation. Sarcomeres in rat neonatal cardiomyocytes that were warmed at 38–42 °C oscillated at both slow (~ 1.4 Hz), Ca^2+^-dependent frequencies and fast (~ 7 Hz), Ca^2+^-independent frequencies. Our high-precision experimental observations revealed that the fast sarcomeric oscillation had high and low peak-to-peak amplitude at low and high [Ca^2+^], respectively; nevertheless, the oscillation period remained constant. Our numerical simulations suggest that the regular and fast rthythm is maintained by the unchanged cooperative binding behavior of myosin molecules during slow oscillatory changes in [Ca^2+^].

## Introduction

The cardiac cycle is synchronized physiologically by oscillatory changes in [Ca^2+^]; in this cycle, cardiomyocytes contract at high [Ca^2+^] and relax at low [Ca^2+^]^1,2^. However, the Frank-Starling law—which specifies that more blood returning to the ventricle causes more blood to be ejected during the next systole—is thought to be dependent on sarcomere structure and independent of [Ca^2+^]^3–8^. The rapid distention of the ventricular myocardium during early diastolic filling is also thought to be a mechanism that does not depend on [Ca^2+^]^9,10^. These facts suggest that the heart rhythm is controlled by both [Ca^2+^]-dependent and [Ca^2+^]-independent regulatory mechanisms.

In fact, spontaneous oscillatory contraction (SPOC) of cardiomyocytes is observed even at constant [Ca^2+^] (~ pCa 6) in cardiomyocytes stripped of their membranes and in myofibrils purified from cardiomyocytes^11–15^. The frequencies of SPOC in cardiomyocytes purified from the hearts of cows, pigs, dogs, rabbits and rats are proportional to the beat frequencies of the corresponding hearts^16^. Living cardiomyocytes treated with ionomycin (a selective Ca^2+^ ionophore) have been found to exhibit SPOC at constant intercellular [Ca^2+^] due to inhibition of Ca^2+^ channels and pumps in the sarcoplasmic reticulum^17,18^. These results suggest that cardiomyocytes exhibit oscillatory contraction even at constant [Ca^2+^] and that this contraction might be coupled to the physiology of the heartbeat in vivo.

In addition to SPOC at constant [Ca^2+^], the characteristics of oscillatory behavior at elevated temperatures in association with time-dependent [Ca^2+^] transients should provide further insights into the mechanism of oscillation and its contribution to heart function. Transient contractions of living adult cardiomyocytes have been detected upon a rapid temperature increase from 36 to 41 °C^19^. In that study, the [Ca^2+^] as measured by a calcium indicator (Fluo-4) was the same at 41 °C as in relaxed cells at 36 °C, suggesting that the contraction system of cardiomyocytes is activated at high temperatures even under conditions of very low [Ca^2+^]^19^. This activation has also been confirmed in an in vitro motility assay using thin filaments reconstituted with α-tropomyosin and troponin^20^. These filaments were found to be able to slide at a rate of approximately 30% of full activity at a temperature of approximately 37 °C even at a low [Ca^2+^]^20^. Adult cardiomyocytes do not show SPOC with temperature jumps, but neonatal cardiomyocytes exhibit oscillatory contraction at high frequencies (6–12 Hz) under conditions of low-frequency Ca^2+^ oscillation (~ 1.4 Hz)^18^. The frequency of 6–8 Hz observed at high temperature is close to the neonatal heart rate, while the frequency of ~ 1 Hz Ca^2+^ oscillation coexisting with the high-frequency contractile oscillation is close to the frequency of spontaneous cardiomyocyte oscillation at 37 °C. The frequency of sarcomeric oscillations (hyperthermal sarcomeric oscillations, HSOs) is elevated at high temperature but rapidly returns to the original frequency when the temperature is lowered, indicating the reversibility of the change in oscillation frequency^18^.

Sarcomeres usually contract strongly and shorten rapidly at high [Ca^2+^]. However, sarcomere lengthening during HSOs is observed at high [Ca^2+^] as well as at low [Ca^2+^]. The occurrence of sarcomere lengthening even at high [Ca^2+^] indicates that the mechanism of SPOC exerts a certain effect on the relaxation of cardiomyocytes in a beating heart with standard time-dependent [Ca^2+^] transients. In previous work, the waveforms of HSOs with frequencies of 7–10 Hz could not be analyzed effectively because the length changes during HSO were observed at a low frame rate of 33 frames per second (fps) and with low spatial precision (~ 8 nm)^18^. The observation of HSOs at higher spatiotemporal accuracy (500 fps and 4 nm) in the present study enabled detailed analysis of the oscillatory behavior of sarcomeres. In addition, the obtained results were integrated with the effect of temperature in our previous numerical model^21^ to simulate the stochastic behavior of the actomyosin ATPase cycle, including binding control by the troponin-tropomyosin complex. HSOs had large and small amplitudes at low and high [Ca^2+^], respectively. The frequencies of HSOs remained constant independent of the amplitudes of the oscillations and of [Ca^2+^]. Analysis with the numerical simulation model suggest that the regular rhythm of HSOs comes from the unchanged cooperative behavior of active myosin molecules under conditions of slow oscillatory changes in [Ca^2+^]. Our work helps clarify the molecular mechanism of diastolic heart failure.

## Results

### Characteristics of HSOs induced by temperature jumps

**Figure 1:**
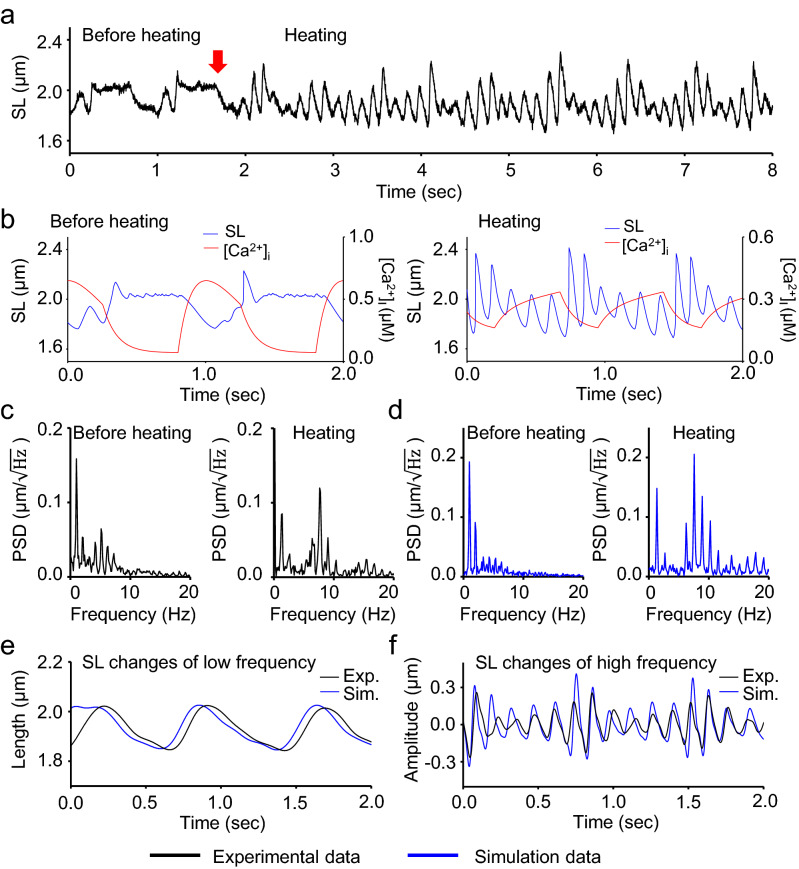
Sarcomere oscillation waveforms before and after HSO occurrence.** (a)** Typical example of an oscillation waveform of sarcomere length (SL) in cardiomyocytes. The infrared laser was activated at the time indicated by the red arrow. HSOs occurred promptly after irradiation. (**b)** Simulation of the oscillation before (left panel) and after the temperature jump (right panel). (**c,d**) Power spectrum of a sarcomere oscillation waveform in the experiment **(c)** and in the simulation **(d)** before (left panels) and after the temperature jump (right panels). (**e,f)** Sarcomere oscillations with frequency ranges of 0 to 3.5 Hz **(e)** and 3.5 to 25 Hz **(f)**. Black lines, experimental data; blue lines, simulation data.

Sarcomeric oscillation in cardiomyocytes was clearly observed even at a frame rate of 500 Hz (Supplementary Video 1; Supplementary Fig. 1, 2). Sarcomere length was measured precisely by fitting the intensity profile of α-actinin-GFP at the Z-line to a parabolic function (Fig. 1a). The oscillation frequency increased from ~ 1 to ~ 7 Hz over a temperature jump from 37 to 41 °C (Fig. 1, Supplementary Video 1). At 37 °C, the predominant power spectral density (PSD) peak of sarcomeric oscillation was at 1.0 Hz, while the minor peak was at 5.1 Hz (Fig. 1c, left). At 41 °C, the predominant peak was found at 7.6 Hz, and a minor peak was found at ~ 1.4 Hz (Fig. 1c, right). The peak frequencies at 41 °C were ~ 1.4 times of those at 37 °C (Fig. 1c). In order to separate the low-frequency and high-frequency oscillation components, the oscillation traces were filtered with a low-pass filter at 3.5 Hz and a bandpass filter between 3.5 (or 3.0) and 25 Hz (Fig. 1e,f and Supplementary Fig. 1). The low-frequency component (1.4 Hz) of HSO was coupled with [Ca^2+^] transients (Fig. 1e)^18^, but high-frequency oscillation was not coupled directly with oscillation of [Ca^2+^] (Fig. 1f). Even at a normal temperature of 37 °C, some high-frequency oscillation was observed (Supplementary Fig. 1). Since the temporal resolution (2 ms) of the measurement was better in this study than in prior work, two subpeaks (6.3 and 9.0 Hz) appeared on opposite sides of the predominant peak (7.6 Hz) at 41 °C. The difference between the subpeak and predominant peak was close to 1.4 Hz, which indicates a slow frequency of oscillation. The subpeaks were produced mathematically via modulation of the amplitude of 7.6 Hz oscillation by 1.4 Hz, likely as the side bands appeared in the amplitude-modified (AM) wave, so that the frequency of the amplitude change of HSOs was 1.4 Hz. This indicates that the period of HSO was constant despite the modulation of the oscillation amplitude by low-frequency oscillation coupled with [Ca^2+^] transients.

**Figure 2:**
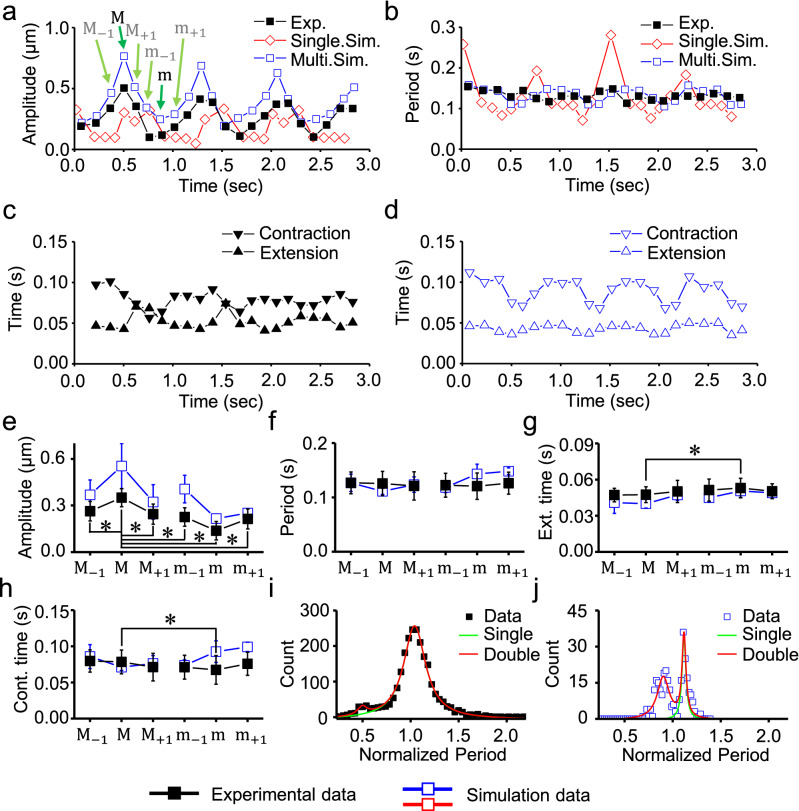
Statistical analysis results for the oscillation characteristics of HSOs. **(a,b)** Changes in the amplitude (**a**) and period (**b**) of the oscillation waveform in Fig. 1f. (**c,d)** Changes in contraction (inverted triangles) and extension time (triangles) for the oscillation waveform in Fig. 1f. The experimental data (**c**) and simulation data (**d**) are shown for the contraction and extension times. (**e–h)** The oscillation cycles where the amplitude reached the maximum (“M-cycle”) and minimum (“m-cycle”) and the cycles before and after the M- and m-cycles were analyzed. (**e**), oscillation amplitude; (**f**), oscillation period; (**g**), extension time; (**h**), contraction time. Significant differences between groups are indicated with an asterisk (*). All pairs of maximum amplitudes and other amplitudes in (**e**) had significant differences. **(i,j)** Histograms of the oscillation periods normalized by the mean periods for the (**i**) experimental data and (**f**) simulation data. The data were fitted to double Lorentz distributions (**i**; R = 0.996**, j**; R = 0.940). Closed symbols, experimental data; open blue symbols, simulation data (**a–j**). The green arrows show specific examples of the labels (**e–h**) for the open blue symbols (**a**). The open red symbols indicate the single-half-sarcomere model simulation data (**a,b**). Black lines, experimental data; blue lines, simulation data.

As shown in Supplementary Video 1, the contraction of one sarcomere was propagated along the myofibril. Changes in the lengths of 5 consecutive sarcomeres in a myofibril were analyzed to understand the propagation (Supplementary Fig. 3). The mean timepoints of the maximum and minimum lengths were shifted gradually from one sarcomere to the next. The length change of the 5th sarcomere was shifted by approximately half the period of the 1st one.

**Figure 3:**
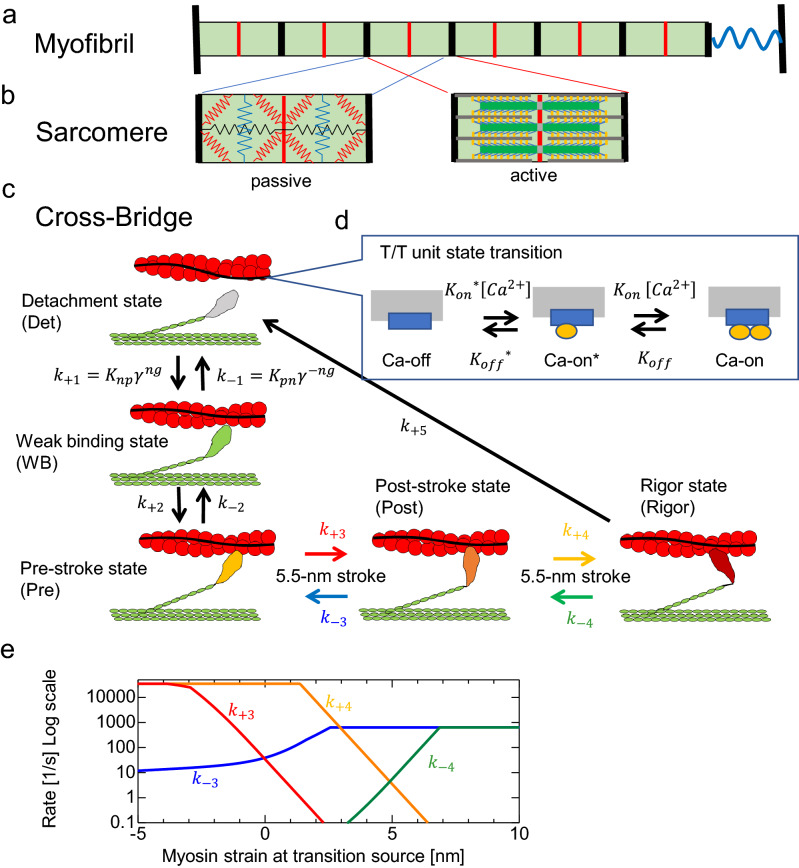
Schematic diagram of the model used to simulate HSOs.** (a)** Myofibril model. The black and red lines indicate the Z and M lines, respectively. The area between the black lines is a sarcomere. The wavy blue line at the right end is an elastic element. **(b)** Sarcomere model. The left panel shows the arrangement of elastic elements, and the right panel shows the arrangement of myosin-derived contractile elements. (**c)** Stochastic state transition model of the actomyosin complex. The active tension was caused by three attached states (Pre, Post, and Rig). The transitions between the Det and WB states were influenced by the state of the T/T unit. (**d)** T/T unit above the myosin head with the coefficients *K*_*np*_ and *K*_*pn*_ and the states of the neighboring myosin molecules determined via the cooperative factors *γ*^*ng*^ and *γ*^*−ng*^ (*γ* = 60). Here, ng (= 0, 1, or 2) is the number of neighboring myosin heads in either the WB state or the bound states (Pre, Post, and Rig). (**e)** The rate constants *k*_+*3*_*, k*_*−3*_*, k*_+*4*_*,* and *k*_*−4*_ for transitions between the bound states are given as functions of the rod strain. The details are given in the “Methods”.

The amplitudes and periods of oscillations were analyzed; the results are shown in Fig. 2a–d. As expected from the amplitude modulation, the oscillation amplitudes of HSOs changed cyclically by a factor of 3–4 (Fig. 2a), while the oscillation periods of HSOs remained nearly constant (Fig. 2b). The oscillation waveform was composed of a combination of contraction and relaxation phases; thus, the oscillation period was the sum of the contraction time and the relaxation time (Fig. 2c). The contraction and relaxation times changed in an oscillatory manner, while the oscillation period was almost constant (Fig. 2b,c).

The oscillation frequency and the amplitude change shown in Fig. 2a were slightly different between cells. Therefore, the features of the oscillation waveforms, including the maximum and minimum amplitudes, were analyzed for five consecutive sarcomeres of the seven cardiomyocytes, for a total of 35 sarcomeres. The single cycles of oscillation where the oscillation amplitude reached the maximum and minimum values were defined as the “M-cycle” and “m-cycle”, respectively. The oscillation amplitude changed significantly (Fig. 2e), while the oscillation period was almost constant (Fig. 2f). Significant differences in the contraction and extension times between the M- and m-cycles were observed (Fig. 2g,h). These changes were confirmed in the four cardiomyocytes obtained from different isolated batches, indicating that the variations in results between cells and between isolated batches is small (Supplementary Fig. 4). These results suggested that the contraction and extension times underwent complementary changes to keep the period almost constant (Fig. 2f–h).

**Figure 4:**
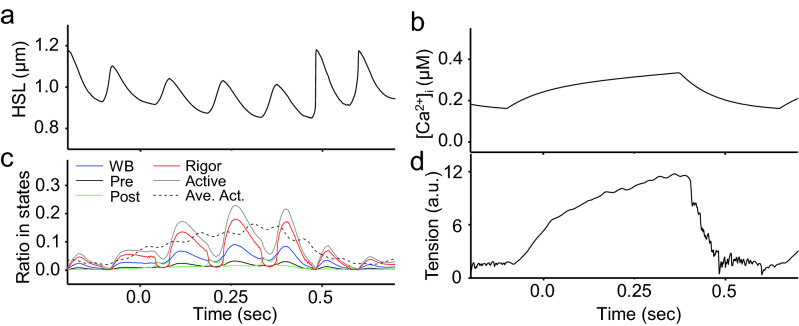
Parameter changes of the model that reproduces HSOs (part 1). The recording time was the same for (**a–d)**.** (a)** Changes in half-sarcomere length. (**b)** Changes in [Ca^2+^]. (**c)** Changes in the number of myosin molecules in each state. Blue, WB state; black, Pre state; green, Post state; red, Rig state. The ratio became 1 when the Det state was included. The sum of the numbers of myosin molecules in the Pre, Post, and Rig states is indicated by “Active (states)” (gray line). The average value of the active myosin of the 40 half-sarcomeres used in the model is indicated by “Ave. Act.” with a black dashed line.** (d)** Tension generated by the active myosin molecules.

In order to understand the period distribution, the oscillation periods measured in the different sarcomeres were normalized by the mean value and are shown in a histogram (Fig. 2i). The histogram was fitted to a double Lorentz distribution with the peaks at a normalized period of 1.0 and at half of this period (0.5) (Fig. 2i). Oscillation waveforms with short periods were rarely observed (3.8% of the analyzed waveforms) (Supplementary Fig. 5; Fig. 2i). Oscillation waveforms with short periods were excluded from the analyses in Fig. 2e–h.

**Figure 5:**
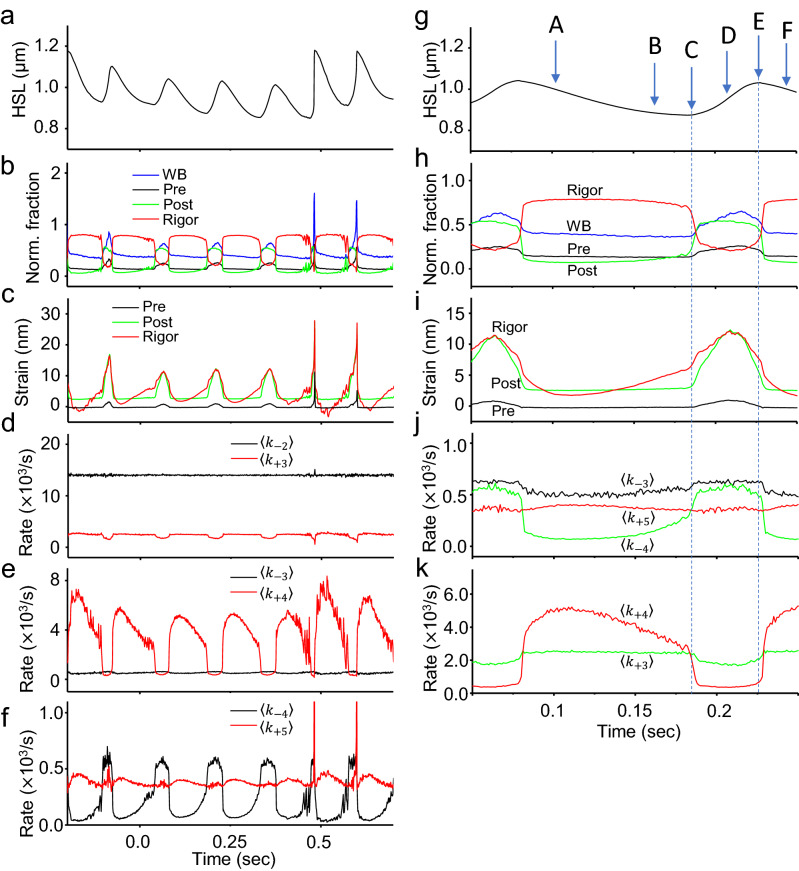
Parameter changes of the model that reproduces HSOs (part 2). The recording time was the same for (**a–f)** and Fig. 4. ** (a)** Changes in half-sarcomere length (same data as in Fig. 4a). (**b)** The numbers of myosin molecules in the different states (Fig. 4c) were normalized by the number of active molecules. The colors are the same as in (**a**). (**c)** Average strain applied to each myosin molecule in each state. The colors are the same as in (**a**). (**d–f)** Rate constant of the state transition of myosin in each state. The vertical axis of the graphs shows the 1/1000 value. The constant for the transition from Pre to Post is < k_+3_ > , and that for the transition from Pre to WB is < k_−2_ > (**d**). The constant for the transition from Post to Rig is < k_+4_ > , and that for the transition from Post to Pre is < k_−3_ > (**e**). The constant for the transition from Rig to Det is < k_+5_ > , and that for the transition from Rigor to Post is < k_−4_ > (**f**). (**g–k)** Enlarged views of **(a–f)** from 0.05 to 0.25 s. The enlarged view of **(d–f)** was reorganized into two panels except for < k_−2_ > . A–F are symbols for explaining the mechanism of HSOs in the text.

### Model simulation of HSOs

To clarify the reason for the constant period, we reproduced the phenomenon by using a single-myofibril model consisting of 40 half-sarcomeres in which a stochastic actomyosin ATPase cycle model with a troponin-tropomyosin complex was imbedded (see Fig. 3 and Methods section). In this model, the tension of each half-sarcomere was computed as the sum of the tensions pulling the thin filaments by the myosin molecules in the three strong binding states (prestroke (Pre), poststroke (Post), and rigor (Rig) states, Fig. 3c). Half-sarcomere shortening is directly reflected by changes in myosin strain in the strong binding states. As a result, the shortening (or lengthening) of a half-sarcomere leads to facilitation of power stroke (or stroke reversal) transitions, which is captured in the model by increases in the rate constants ${k}_{+3}$ and ${k}_{+4}$ (or ${k}_{-3}$ and ${k}_{-4}$) (Fig. 3e). The model used in this simulation is based on a previous model but newly assumes an effect of temperature^20^. A temperature increase from 37 to 41 °C was modeled by multiplying the rate constants of all the transitions by a factor of 7/4 and by multiplying the rate constant from the detachment (Det) state to the weak binding (WB) state by an additional factor of 2 (${K}_{np}$ in ${k}_{+1}$, Fig. 3c) under the assumption that heating reduces the hindrance of binding points by tropomyosin. Increasing the [Ca^2+^] sensitivity of regulatory proteins allowed the generation of a WB state even at a low [Ca^2+^], and increasing the rate constant of the crossbridge cycle shortened the period of the HSOs.

The model reproduced the sarcomeric oscillations well at 37 °C and 41 °C (Fig. 1b,d–f). HSOs were also observed at an intermediate temperature of 39 °C (Supplementary Fig. 1d). While the amplitudes of HSOs fluctuated, the characteristic of a near-constant oscillation period was reproduced (Fig. 2a,b,d–h,j). In greater detail, the contraction time when the HSO amplitudes reached the minimum value differed slightly in the simulation results (Fig. 2h). As a result, although the width of the frequency distribution of the oscillation period was consistent between the experimental results and the simulation results, the frequency distribution of the oscillation periods in the simulation results was bimodal (Fig. 2j).

To understand the molecular mechanism of HSOs, we analyzed the number of myosin molecules in each state, the reaction rates and the tension. The tension change was coupled with the slow oscillation of [Ca^2+^] but hardly with high frequency oscillation (Fig. 4a,b,d). The concentration of myosin in the active states (Pre, Post and Rig) changed cyclically, while the active myosin averaged for half-sarcomeres changed smoothly (Fig. 4c). This indicates that the length changes in the 40 half-sarcomeres were not synchronized. The fraction of myosin in each state normalized to the active myosin showed a constant pattern almost independent of [Ca^2+^] (Fig. 5b). The strain and the reaction rates also changed with almost constant patterns (Fig. 5d–f).

To understand the phenomenon of cyclic oscillation, we expanded one cycle at intermediate [Ca^2+^], as shown in Fig. 5g–k. During the shortening phase, the second-stroke and stroke reversal rates of < k_+4_ > and < k_−4_ > (Post ← Rig) gradually decreased and increased, respectively, with an increase in average strain in the Rig state (A to B in Fig. 5g–k). The shortening velocity of sarcomeres was slowed by an increase in the myosin strain (B to C). The population of myosin molecules with high strain became large at the end of the shortening period (C), and then an avalanche of power stroke reversals < k_−4_ > of the highly strained myosin was triggered by a small increase in the sarcomere length, resulting in fast sarcomere elongation (C to D in Fig. 5g–k). In the process from D to F, myosin strain at the Rig and Post states started to decrease, and then the reverse process from C to A occurred.

**Figure 6:**
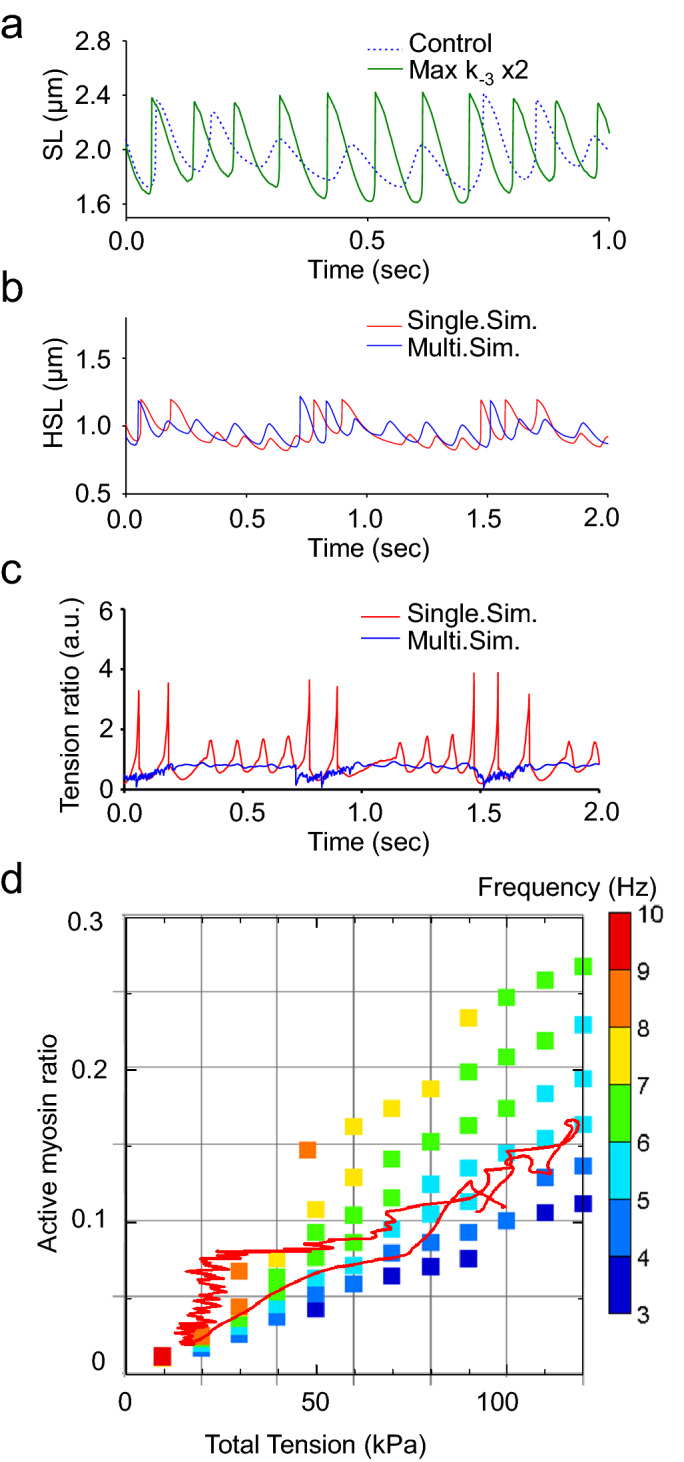
Simulation results for HSOs with different assumptions. **(a)** Time series of changes in sarcomere length. The changes in sarcomere length that occurred when the upper limit of the k_−3_ value was changed from 100 to 200 are shown with green lines. The sarcomere length changes that occurred when the upper limit of the value of k_3_ was kept at 100 are shown with blue broken lines. (**b)** Time series of changes in half-sarcomere length. The results for the single-half-sarcomere model are shown in red, and those for the 40-half-sarcomere model are shown in blue. (**c)** Time series of changes in the average tension applied to one myosin molecule forming a crossbridge. The results for the single-half-sarcomere model are shown in red, and those for the 40-half-sarcomere model are shown in blue. (**d)** Comparison results for the single-half-sarcomere model under constant external tension and [Ca^2+^] and the HSO reproduction model (myofibril model with [Ca^2+^] changes) under a changing external tension and myosin binding ratio. The red line is the trajectory of the HSO reproduction model. (**d**) was created using GraphR (https://www.graph-project.com/) and Sma4Win (https://hp.vector.co.jp/authors/VA002995/soft/sma4win.htm).

It was important to avoid using an excessive upper limit of stroke reversal rate of < k_−3_ > at high strain to appropriately determine the amplitude and period of oscillation (Fig. 3e; Supplementary Formula (S15)). This < k_−3_ > value became the rate-determining factor, and a certain amount of myosin in the Post state remained during the development of the half-sarcomere, producing a slow extension speed resistant to tension and a low HSO amplitude (C in Fig. 5g–k). Myosin in the WB state increased beginning in the early stage of half-sarcomere extension (D in Fig. 5g–k). Since myosin in this WB state had no strain on average, it quickly moved to the Pre state without strain on average according to the transition rate < k_+2_ > (WB → Pre). Most of the myosin that transitioned to the Pre state generated tension by transitioning to the Post state according to the transition rate < k_+3_ > (Pre → Post) and suppressed the extension of the half-sarcomere (Fig. 3e; D in Fig. 5g–k). When the upper limit of the reverse rate *k*_-3_ was changed by double the value, the period became constant, and the amplitude was large at high [Ca^2+^], exhibiting a trend inverse to that of the experimental oscillation (Fig. 6a). Therefore, the power stroke reversals (Pre ← Post) were key reactions in sarcomere lengthening and period constancy.

To investigate the impacts of mechanical interactions between the half-sarcomeres in the myofibril on HSOs, we also created a single-half-sarcomere simulation of HSOs and compared the results with those for the myofibril model containing 40 half-sarcomeres. In the single-half-sarcomere model, the period of oscillation was no longer constant (Figs. 2a, b and 6b). In this model, the tension necessarily changed as the length changed, but in the myofibril model with 40 half-sarcomeres, tension was equivalent through all half-sarcomeres and changed in proportion to the total length change of the 40 half-sarcomeres. The contraction of half-sarcomeres was propagated along the myofibril, consistent with the experimental results (Supplementary Fig. 3). Shortening of some half-sarcomeres was compensated by lengthening of others due to the phase shifts, such that the total length and tension changed slowly with changes in [Ca^2+^] (Fig. 4b,d). This difference in the relationship (or frequency) between half-sarcomere length and tension changes was the main reason for the different behaviors (Figs. 4c, 6c). In other words, the multisarcomere array contributed to collective and asynchronous contraction by avoiding the tension oscillation.

To explore the further necessary conditions for the period constancy of HSOs, we also simulated the isotonic oscillation of the single-half-sarcomere model with various external tensions and [Ca^2+^] values (Supplementary Fig. 6a, b). We found that the frequency decreased (or increased) as the external tension (or [Ca^2+^]) increased under constant [Ca^2+^] (or external tension) (Supplementary Fig. 6c, d). The HSO trajectory on the plane of external tension and the binding myosin ratio for a single half-sarcomere in the myofibril model remained mostly within the 5–7 Hz zone (red line in Fig. 6d). This indicates the importance of the myofibril property that imposes a boundary condition on each half-sarcomere such that the relationship between the binding ratio and tension is preserved to establish period constancy.

## Discussion

HSOs were measured at high spatial and temporal resolutions of 500 Hz and 4 nm to understand the fundamental mechanism of the oscillations. HSOs were composed of oscillations of two frequencies: [Ca^2+^]-dependent slow oscillations and [Ca^2+^]-independent fast oscillations (see Fig. 1C from^18^). For fast oscillations, the period of each cycle was kept constant (Fig. 2). The oscillation simulation revealed the mechanism of the period constancy (contraction rhythm homeostasis): the regular periods were maintained by the unchanged cooperative binding behavior of myosin molecules under slow oscillatory changes in [Ca^2+^].

Regular HSOs were induced when neonatal cardiomyocytes were heated (39–41 °C). Surprisingly, partial HSOs were also observed at ~ 37 °C before heating (Fig. 1a,c; Supplementary Fig. 1). In the simulated oscillation before heating, HSOs appeared with transient changes in [Ca^2+^] from high to low concentrations but did not appear at low concentrations (Fig. 1b). At high temperature, the [Ca^2+^] sensitivity of troponin/tropomyosin (T/T) increased such that HSOs were observed after heating even at low [Ca^2+^]^20^ (Fig. 3c, d). These results suggest that HSOs are present to some extent at physiological body temperatures, at least in neonatal rats^24^, and fully prevail at elevated temperatures due to high activation even at low [Ca^2+^].

In a previous work^18^, amplitude changes synchronized with [Ca^2+^] oscillations were observed (Fig. 2a), but the period of each cycle of oscillation was not measured because of a lack of temporal resolution. Here, the contraction and extension times in each cycle were measured separately and changed in a slow oscillation cycle coupled with [Ca^2+^] changes (Fig. 2b). The total periods of contraction and extension times, however, remained constant (Fig. 2b,c,f). The constant period or frequency was ensured by the sharp peak in the power spectrum (Fig. 1c). The constant frequency of 7 Hz was very close to the frequency of the mouse heartbeat^22^, possibly indicating that the heartbeat, regulated by pacemaker cells, is set to the frequency of HSOs originating from contractile proteins in sarcomeres. This idea is supported by the result that the frequencies of SPOC of cardiomyocytes purified from several species are proportional to the heartbeat frequencies of the corresponding hearts^16^.

The amplitude (~ 0.2 µm) and maximum sarcomere length (~ 2.0 µm) of the slow component of oscillation (Fig. 1e) were consistent with those (0.25 µm and 1.9 µm) of the live adult mouse heart^23^. However, the contraction amplitude of 0.6 µm and maximum sarcomere length of 2.4 µm of HSOs (Fig. 1) are larger than those. The reason for the discrepancy is that most of the active myosin in a half-sarcomere is dissociated from actin (approximately 0.45 s in Fig. 4c) so that the half-sarcomere is lengthened considerably by other contracting sarcomeres. In addition, the force (~ 0.75 pN per titin molecule) of neonatal titin during relaxation is only one-third of that (2 pN) of adult titin at 2.4 µm^24^. This is calculated to be 4.5 pN per half myosin filament because 6 titin molecules bind per half myosin filament^25,26^. The titin force of 4.5 pN is only about half of  the force (8 pN) generated by single myosin molecules^27^ so that a small imbalance of numbers of active myosin between sarcomeres explains the long sarcomere length.

To understand the molecular mechanism of period constancy, we reproduced HSOs with the numerical single-myofibril simulation model (Fig. 3) proposed in our previous work^21^. Several reaction rates in the crossbridge cycle in the model are known to change with temperature. The binding affinity of troponin and tropomyosin for actin filaments decreases with increasing temperature, and then the sensitivity of [Ca^2+^] increases^20,30–32^. The speed of the crossbridge cycle of myosin and the stroke reversal rate in skeletal muscle myosin increase with increasing temperature^20,33–35^. When these changes were applied in the model simulation, the HSOs were reproduced reasonably well; the amplitudes of HSOs changed greatly while the period remained constant (Figs. 1 and 2).

What is the molecular mechanism of period constancy? The number of myosin molecules in active states in one half-sarcomere compared with a myofibril of 40 half-sarcomeres changed at the frequency of HSOs (Fig. 4c). The peak number of active myosin molecules in a single cycle of HSOs changed in parallel to the tension change. The number of myosin molecules in each state was normalized to the total number of active myosin molecules (Pre, Post, Rig); however, it changed independently of tension with a constant pattern and peak value (Fig. 5b). The mean strain also showed an almost constant pattern (Fig. 5c). These constant patterns of normalized myosin fraction in the active states (or strong binding states) and the mean strain established the period constancy of HSOs. In other words, the regular rhythm of HSOs comes from the cooperative behavior of active myosin molecules, which is independent of the total number of active myosin molecules during slow oscillatory changes in [Ca^2+^].

The next question was why a high-frequency cycle was induced. The high-frequency and [Ca^2+^]-independent oscillation was reproduced with sarcomere lengthening caused by an avalanche of stroke reversals from the Rig to the Post state (*k*_−4_) and from the Post to the Pre state (*k*_−3_). At the end of the shortening phase, the population of myosin with high strain was large, and then an avalanche of power stroke reversals and < k_−4_ > could be easily triggered by minor sarcomere lengthening (C to D in Fig. 5g–k). The tension was reduced by these stroke reversals, and then the sarcomeres began to lengthen. Therefore, power stroke reversals are key reactions in the cycle of HSOs^21,28^. Notably, ATP was not utilized in the reverse reaction so that the HSOs are highly efficient.

Our final question was why the amplitude of HSOs was high at low [Ca^2+^] (Fig. 1b,f). There were very small numbers of myosin molecules in the active state at low [Ca^2+^] and at the end of shortening (~ 0.49 s in Fig. 4c, C in Supplementary Fig. 7). Given the substantial fraction of myosin in Rig, there was a small decrease in the number of myosin molecules in Rig undergoing stroke reversals (Post ← Rig). Then, the average strain in the Rig state increased very steeply to compensate for the strain reduction during the transition to the Post state through stroke reversal (C to D in Supplementary Fig. 7). This accelerated the increases in Post myosin and then in Pre and WB myosin. Then, an avalanche of power stroke reversals (< k_−4_ > and < k_−3_ >) was triggered by a small increase in the sarcomere length (D in Supplementary Fig. 7). Since the number of active myosin molecules was very small at low [Ca^2+^], the avalanche effect was more prominent at low [Ca^2+^] than at high [Ca^2+^], and then fast and extensive lengthening was induced for a short period (D to E in Supplementary Fig. 7). The assumption of cooperativity, which holds that neighboring myosin heads tend to become active, was also considered to reduce the number of active myosin molecules and accelerate the extension (Fig. 3c, d; D in Supplementary Fig. 7)^21,28^.

Here, we consider the importance of the molecular mechanism of HSOs to the physiological function of heartbeat. The frequency of heartbeats in resting mice is related to that of the HSOs, as mentioned above. It is reasonable to propose that the molecular function of the constant period (frequency) of HSOs is associated with stabilization of heartbeat frequency. The left ventricle of the heart dilates rapidly in early diastole even under conditions of slow [Ca^2+^] decreases and low ventricular pressure. The myocardial contraction system in the left ventricle must rapidly relax under these conditions. Since temperature is higher in the left ventricle than in other organs because of high energy consumption^29,36^, HSOs may occur. The rapid lengthening observed in HSOs would aid in rapid relaxation of the left ventricle (Fig. 5d–k). From the perspective of efficiency, the energy consumption in the left ventricle should be preserved because no ATP is utilized during the stroke reversal (Fig. 5g–k). HSOs are likely manifestations of the nature of rapid ventricular diastolic relaxation and efficient cycles of contraction and relaxation^9,10,22^. These results suggest that HSOs contribute at least to the essential features of the neonatal heart, such as stable rhythmic contraction and efficient circulation.

## Materials and methods

### Cell preparation

This research was approved by the Animal Experiment Committee of the Faculty of Science at the University of Tokyo and was conducted according to the Animal Experiment Implementation Manual of the University of Tokyo.

Wistar rats (one day old) were purchased from Sankyo Labo Service Corporation (Tokyo, Japan). Ten Wistar rats (one day old) were used in one cell isolation batch. Immature cardiomyocytes were harvested from 1-day-old rats and cultured in medium containing a mixture of Dulbecco's modified Eagle’s medium and Nutrient Mixture F-12) DMEM/F-12, HEPES, Life Technologies), 10% fetal bovine serum, 100 U/ml penicillin and 100 U/ml streptomycin (Life Technologies). The cardiomyocytes were transfected with the pAcGFP-actinin plasmid^17,18^. The cardiomyocytes were observed one day after transfection.

### Microscopic system

The optical system used was similar to that used in our previous work, with some modifications^17,18^. Briefly, an inverted microscope (IX-70, Olympus) was equipped with a high-sensitivity camera (iXon 3 860, iXon Ultra, Andor Technology) and an oil immersion objective (UPlanSapo 100 XO, Olympus). The vibration noise of the microscope was reduced by making the microscope stage rigid and optimizing the performance of a vibration-free table. The size of a pixel on the camera corresponded to 150 nm in the sample. The frame rate was adjusted to 500 fps for high-temporal-resolution analysis. The temperatures of the glass slides seeded with cardiomyocytes were controlled to 37.0 ± 0.2 °C by a thermostatically controlled incubator on the sample stage (INUG 2—ONICS, Tokai Hit Co.). A 1550 nm laser (FPL 1055 T, Thorlabs) was used as a heat source to enable rapid alteration of temperature in the vicinity of the cardiac myocytes being monitored. A 488 nm laser (FITEL HPU50211 (Blue), Furukawa Electric) was used as an excitation light source for fluorescence observation of AcGFP-actinin.

### Data analysis

The intensity profiles of the fluorescence images obtained from the camera were acquired with Solis software (Andor Technology) and analyzed with ImageJ (National Institutes of Health). The sarcomere length was measured precisely by fitting the intensity profile of α-actinin-GFP at the Z-line to a parabolic function^17,18^. The standard deviation of sarcomere length was ~ 4 nm at a frame rate of 500 Hz. Fast Fourier transform (FFT) processing with a Hamming window was performed with LabChart 7 software (ADInstruments) to obtain the power spectrum density. The oscillation components < 3.5 Hz, and the oscillation components from 3.5 to 25 Hz were obtained by low-pass and bandpass filters with LabChart 7 software (ADInstruments). Histograms were created with graphics software (OriginPro 9J, OriginLab Corporation).

### Simulation

In this study, we applied the numerical single-myofibril model (Fig. 3) proposed in our previous work^21^, with some modification of parameters. The details are given in the Supplementary Materials. Here, we present an outline. The myofibril model consisted of 40 half-sarcomeres (Fig. 3a). Each half-sarcomere had passive longitudinal and lateral elasticities, and alignment elasticity existed along the lateral widths between neighboring half-sarcomeres (Fig. 3b left). The longitudinal active stress (Fig. 3b right) was computed from a Monte Carlo crossbridge model (Fig. 3c). In each half-sarcomere, 4096 filament pairs consisting of thin and thick filaments were imbedded, and the active tension was computed as the sum of the pulling tensions imposed on all the thin filaments, where 38 myosin motors were arranged in each one-dimensional filament pair model. We used a [Ca^2+^] profile similar to that observed in our previous work^18^ before heating. The [Ca^2+^] profile after heating was adjusted so that the amplitudes of HSOs were reproduced. The influence of [Ca^2+^] was taken into account in the state transition of the T/T unit (Fig. 3d), which affected the rate constant ${K}_{pn}$ of the transition from the Det state to the WB state in the crossbridge model. The factors ${\gamma }^{ng}$ and ${\gamma }^{-ng}$ with $\gamma =60$ represented the strong nearest-neighbor cooperative properties, where $ng$ was determined from the states of two neighboring crossbridges. Namely, the value of *ng* (= 0, 1, or 2) represented the number of neighboring myosin heads in the WB state or the three binding states (Pre, Post, Rigor). In the binding state, the rate constants ${k}_{\pm 3}$ and ${k}_{\pm 4}$ are given as functions of the myosin rod strain (Fig. 3e). The myosin rod strain was incremented (or decremented) at the power stroke (or the stroke reversal) transition by the stroke distance (5.5 nm). Furthermore, the half-sarcomere length changes were also directly reflected in the changes in the rod strains of the binding myosin molecules in each half-sarcomere model. The influence of heating was modeled by the changes in two parameters. First, all the rate constants for the crossbridge cycling and state transitions of the T/T unit were multiplied by a factor of 7/4. Second, in addition to the total acceleration of the cycle, the rate constant for the transition of the Det state to the WB state (${K}_{np}$) was multiplied by a factor of 2 under the assumption that heating relaxes the hindrance of binding sites by tropomyosin.

### Statistics

All statistical tests were performed with a library of statistical software tools (R language). The normality and homoscedasticity of the amplitudes, periods and times shown in Fig. 2e–h were tested with Kolmogorov–Smirnov and Bartlett tests. The data that satisfied both normality and homoscedasticity assumptions were tested with a multigroup test (Dunnett's test). If the data were not satisfied well, another multigroup test (Steel's test) was conducted. The references in Dunnett’s and Steel's tests were the oscillation amplitude, the contraction and extension times and the sum of these times in a waveform in which the oscillation amplitude became the maximum. The significance level for all statistical analyses was 5%. The error bars in all figures indicate the standard deviations.

## Supplementary information

Supplementary Video 1.

Supplementary Information 1.
