# Supplementary material for: Mechanism of contraction rhythm homeostasis for hyperthermal sarcomeric oscillations of neonatal cardiomyocytes
Source: Sci Rep. 2020 Nov 24;10:20468. doi: 10.1038/s41598-020-77443-x (PMC7687892; doi:10.1038/s41598-020-77443-x)
Supplement: Supplementary file 2 — Supplementary Information 1. [file 41598_2020_77443_MOESM2_ESM.pdf]

Supplementary information

Mechanism of Contraction Rhythm Homeostasis for Hyperthermal Sarcomeric Oscillations of Neonatal Cardiomyocytes

Seine A. Shintani, Takumi Washio and Hideo Higuchi

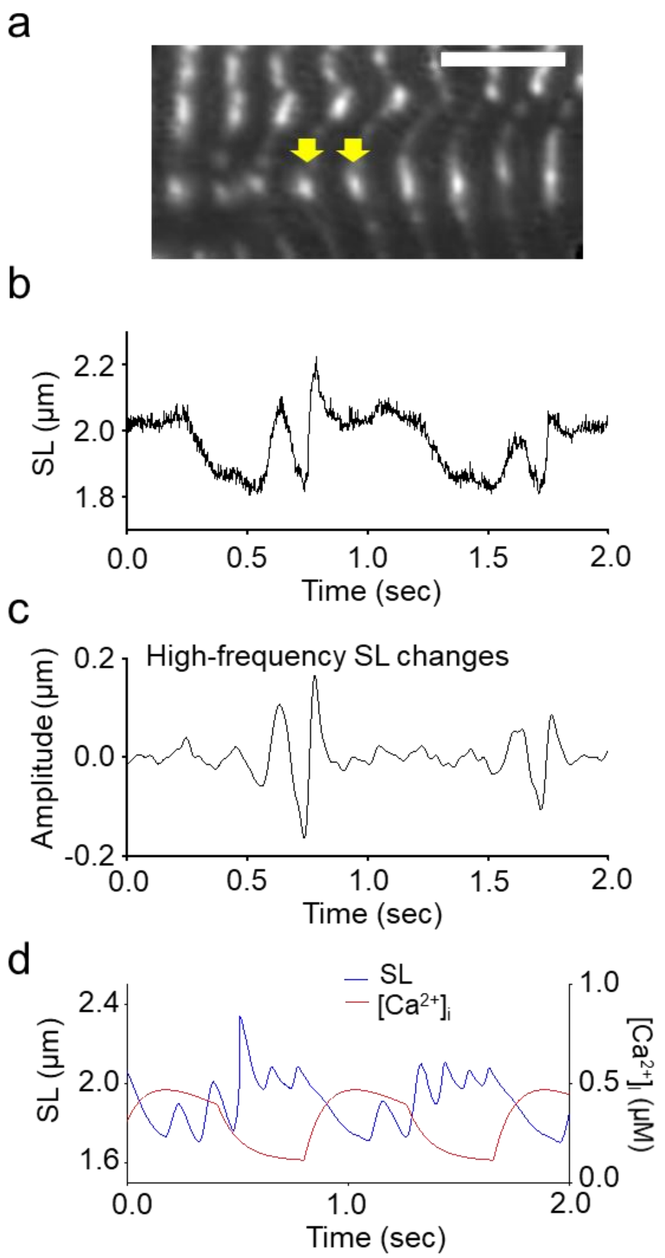

Supplementary Figure 1 | HSO component of sarcomere oscillation at 37 and 39 °C

a. Fluorescence image of cardiomyocytes used for measurement of sarcomeres. **b**. The sarcomere lengths (SL) are those used in Figure 1a at 37 °C and were measured as the distance between the bright lines indicated by the yellow arrows. **c.** High-frequency (3 to 25 Hz) component of the SL change in (**b)** extracted by bandpass filter. **d** Simulation results at 39 °C using parameters between those of 37 °C and 41 °C. The blue line is the sarcomere length, and the red line is the change in calcium concentration. All the rate constants for the crossbridge cycling and state transitions of the T/T unit were multiplied by a factor of 11/8, and the rate constant for the transitions of the Det state to the WB state was multiplied by a factor of 1.5.

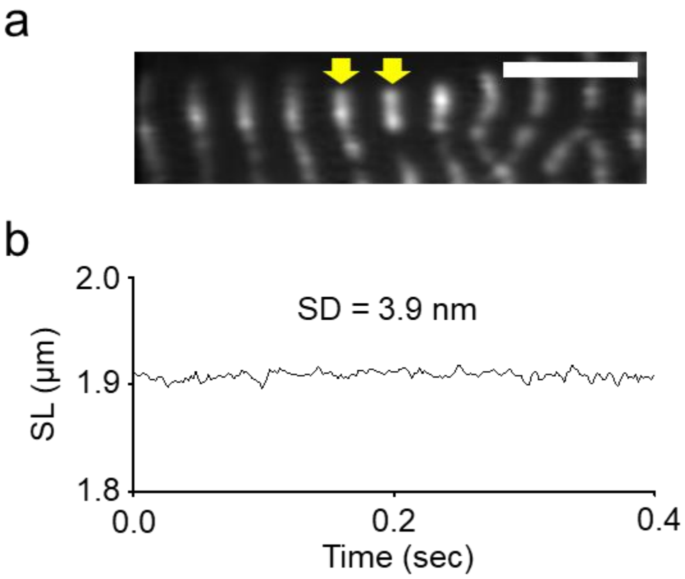

Supplementary Figure 2 | Evaluation of sarcomere length (SL) at nanometer resolution

a Fluorescence image of cardiomyocytes. **b**. The SL was measured as the distance between the bright lines indicated by the yellow arrows. **b** Resting SL between spontaneous beats at 36 °C. The standard deviation (SD) of the SL from zero to 0.4 seconds was 3.9 nm.

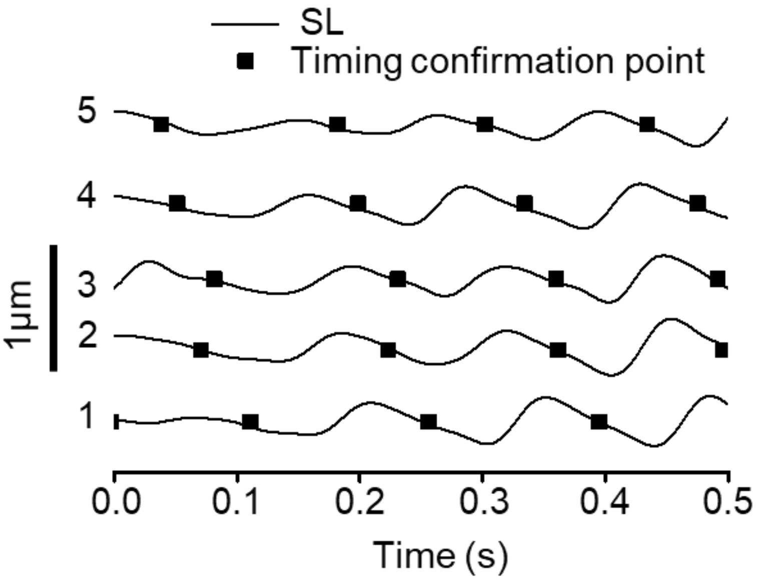

Supplementary Figure 3 | Difference in the timing of oscillations in nearby sarcomeres.

Time series data of SL changes in 5 consecutive adjacent sarcomeres in the HSO state. The midpoint between the timepoint when the sarcomere length reaches the maximum value and the next timepoint when the sarcomere length reaches the minimum value is defined as the timing confirmation point. This timing confirmation point is marked with a black square.

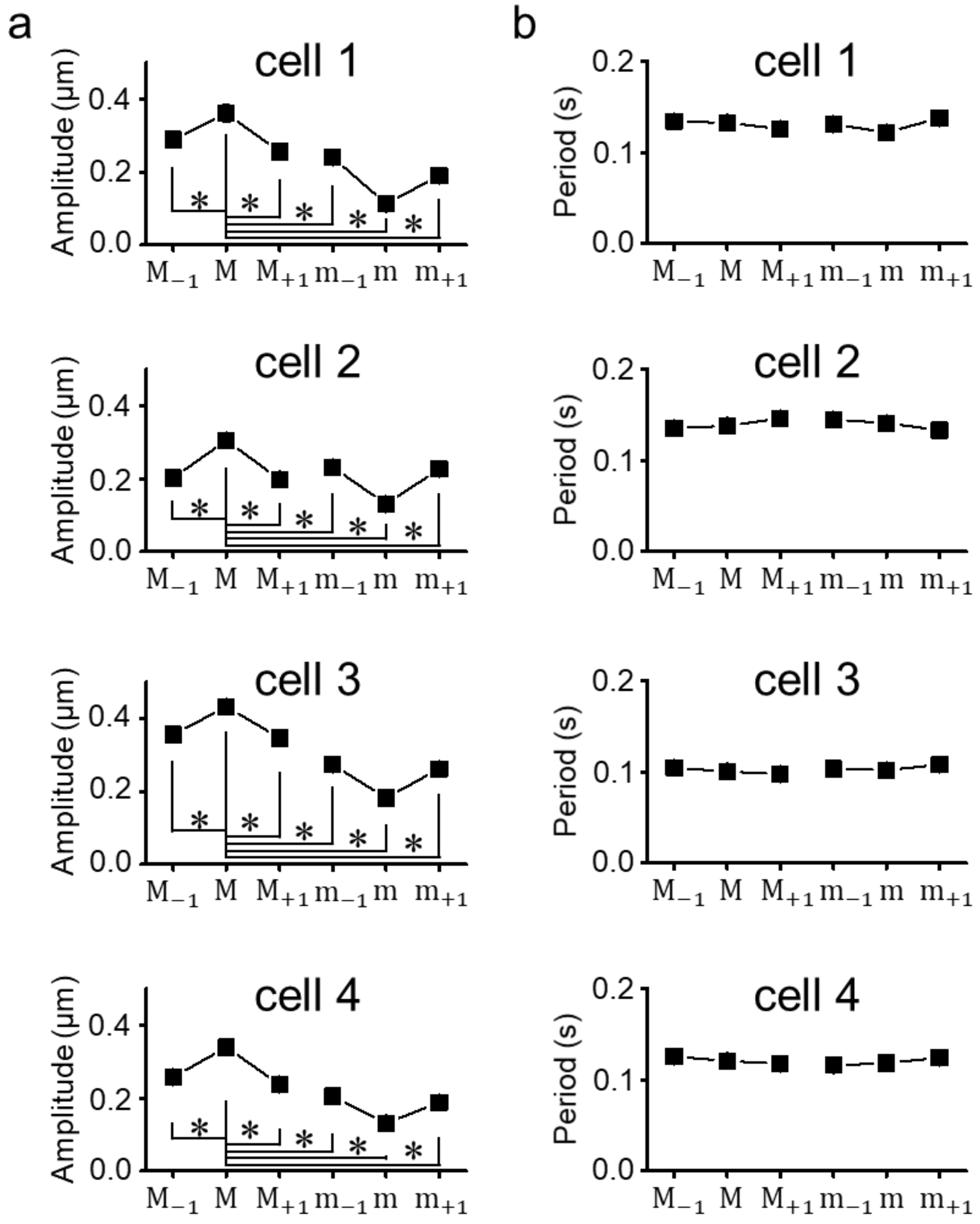

Supplementary Figure 4 | Changes in oscillation amplitude and period of HSOs in each cardiomyocyte

Results of the analysis of Figure 2e, f performed on 5 adjacent sarcomeres of each of 4 cardiomyocytes collected from different rat individuals. Shown are the (**a**) oscillation amplitude and (**b**) oscillation period.

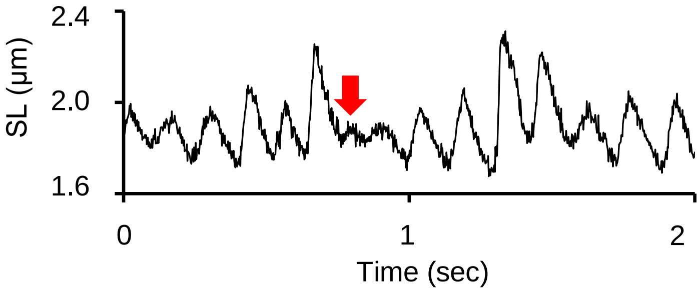

Supplementary Figure 5 | Half-period appearing in HSOs

Time series of sarcomere length during HSOs. The waveform indicated by the red arrow is a half-period of a normal HSO waveform. The frequency of occurrence of this half-period was rare, as indicated by the area ratio of the histogram in Figure 2i.

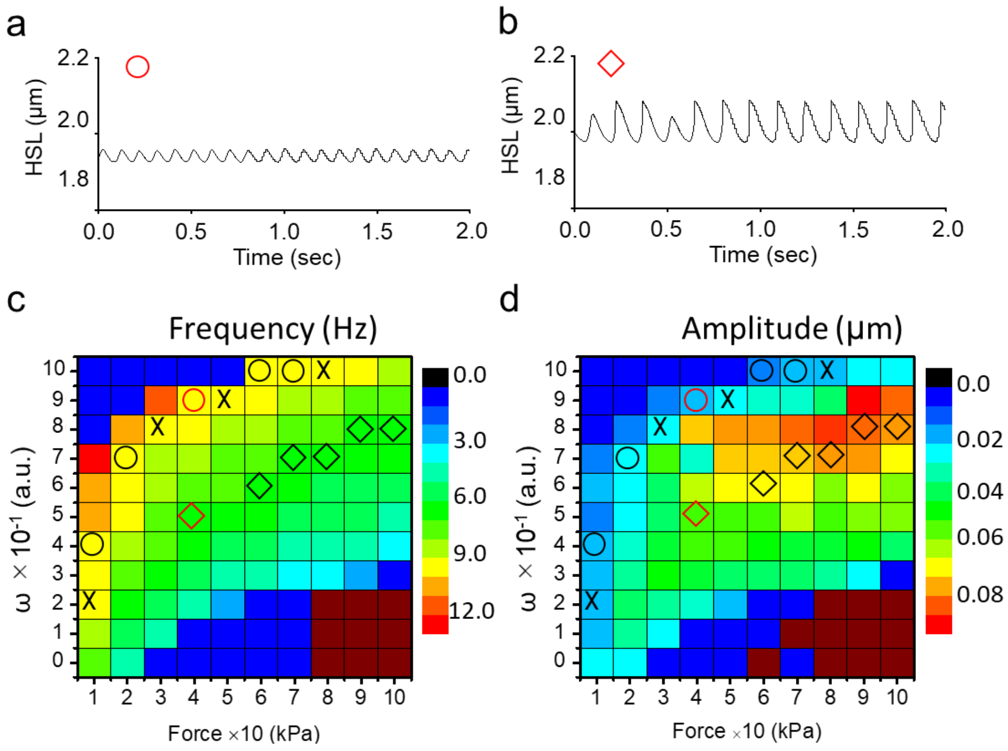

Supplementary Figure 6 | HSOs in a half-sarcomere model under constant external force and [Ca^{2+}]

a, b Waveform of the half-sarcomere length (HSL) model under constant external force and [Ca^{2+}]. (**a**) The [Ca^{2+}] was 1.14 μM, and the external force was 40 kPa. (**b**) The [Ca^{2+}] was 0.39 μM, and the external force was 40 kPa. **c, d** Frequency (**c**) and amplitude (**d**) of HSL oscillation with changing force and [Ca^{2+}]. ω is a parameter for the change in [Ca^{2+}] given by the equation $[Ca^{2+}] = exp\big((1-\omega)log[Ca^{2+}]_{min} + \omega[Ca^{2+}]_{max}\big)$, where $[Ca^{2+}]_{min} = 0.1\,\mu M$ and $[Ca^{2+}]_{max} = 0.4\,\mu M$. The circles, crosses, and diamonds are specific examples of the same frequency. The sarcomere oscillations at the red circle and diamond are shown in (a) and (b), respectively. (c, d) were created using OriginLab OriginPro 9.0 (https://www.originlab.com) and Microsoft Office PowerPoint 2016 (https://www.office.com).

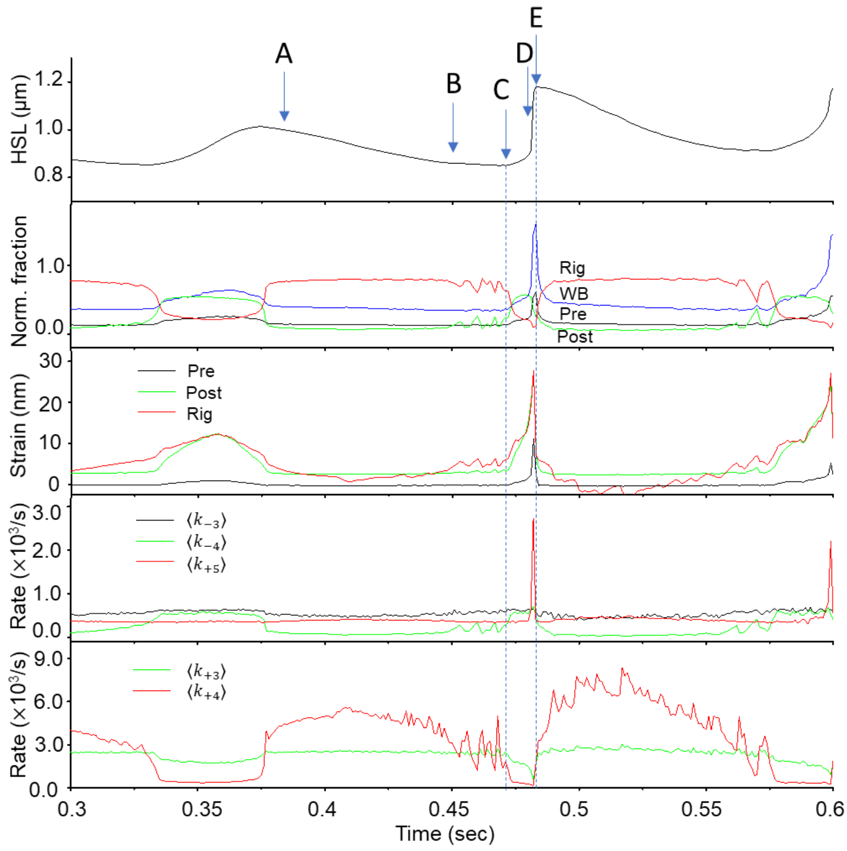

Supplementary Figure 7 | Enlarged view of the simulation results for the maximum HSO amplitude

Enlarged view of Figure 5 (a-f) from 0.05 to 0.25 seconds. Panels g-i were reorganized into two panels except for < k_{-2}>. A-E are symbols for explaining the mechanism of HSOs in the text.

Supplementary Video 1 | HSOs of rat neonatal cardiomyocytes measured with high temporal resolution

Spontaneously beating neonatal cardiomyocytes showing HSOs upon heating. Epifluorescence observation. Objective lens, 60×. Video speed, 500 fps. Scale bar, 5 μm.

Myofibril Model

In the passive elastic model, we assumed only transversely isotropic deformation for each half-sarcomere for simplicity, and we applied the half-sarcomere deformation energy per unit volume in the unloaded condition with the following equation:

$$\psi(\lambda,\mu)=\frac{1}{2}k_{LL}(\lambda-1)^2+k_{TT}(\mu-1)^2+\frac{1}{2}k_{LT}R(\lambda,\mu)^2+L_M(\lambda). \qquad \text{(S1)}$$

Here, λ and μ are stretches in the longitudinal and transverse directions, respectively.
The first and second terms are the deformation energies associated with the longitudinal and transverse elasticities of the half-sarcomere, respectively. Each of them is determined from the strain solely in the associated direction. The third term is a weak penalty term associated with an inverse SL-LS relationship represented as $R(\lambda,\mu)=0$. k_{LT} is a parameter that represents the sarcomere stiffness associated with this penalty. Based on the experimental data for contracted muscle [Ertbjerg2017, Konhilas2002], we adopted a linear function:

$$R(\lambda,\mu)=\lambda-1+2\beta_R(\mu-1), \qquad \text{(S2)}$$

where $\beta_R=2$, $k_{LL}=10\,\text{kPa}$, $k_{TT}=20\,\text{kPa}$, and $k_{LT}=1\,\text{MPa}$. These parameters were adjusted to reproduce the experimental data. Regarding the last term, L_M is the penalty function that prevents extraordinary shortening and stretching from the presence of the thick filament and the filamentous protein called titin connected with the Z-disc. L_M is represented as follows:

$$L_M(\lambda)=\begin{cases} c_{L\min}\dfrac{(\lambda-\lambda_{\text{Lmin}})^4}{4}, \lambda\le\lambda_{\text{Lmin}} \\ 0, \qquad \lambda_{\text{Lmin}}<\lambda<\lambda_{\text{Lmax}}, \\ c_{L\max}\dfrac{(\lambda-\lambda_{\text{Lmax}})^4}{4}, \lambda\ge\lambda_{\text{Lmax}} \end{cases} \qquad \text{(S3)}$$

where $c_{L\min}=5\text{GPa}$, $\lambda_{L\min}=0.87$, $c_{L\max}=100\text{MPa}$, and $\lambda_{L\max}=1.25$ [Washio2016].

We also introduced the deformation energy per unit volume in the unloaded condition for the difference in lattice spacing between the adjacent half-sarcomeres, as follows:

$$\varphi_i(\mu_i,\mu_{i+1}) = \begin{cases} k_{MM}(\mu_i - \mu_{i+1})^2, i = 1,3,\cdots \\ k_{MZ}(\mu_i - \mu_{i+1})^2, i = 2,4,\cdots \end{cases} \tag{S4}$$

Here, μ_i and μ_{i+1} are the transverse stretches of the left and right half-sarcomeres, respectively. The half-sarcomeres are separated by the M-band in the upper case, while they are separated by the Z-disc in the lower case. Because Telley et al. [Telley2006] observed a clear difference in the transfer times of half-sarcomere lengthening over these interfaces, we applied different stiffness parameters of $k_{MM} = 200$ kPa and $k_{MZ} = 50$ kPa. These parameters were adjusted to reproduce the experimental propagation velocity of the lengthening wave.

Under the above deformation energies, the total longitudinal contractile tension in the ith half-sarcomere is given by

$$T_{L,i} = \gamma_L \dot{\lambda}_i + k_{LL}(\lambda_i - 1) + k_{LT}R(\lambda_i,\mu_i) + \frac{\partial L_M}{d\lambda}(\lambda_i) + T_{act,i}. \tag{S5}$$

Here, $\gamma_L = 10\text{Pa}\cdot\text{s}$ is the longitudinal friction coefficient [Washio2017], and $T_{act,i}$ is the active contractile tension generated by the actomyosin complexes (given later). The total transverse contractile tension in the ith half-sarcomere is given by

$$T_{T,i} = \gamma_T \dot{\mu}_i + k_{TT}(\mu_i - 1) + \beta_R k_{LT} R(\lambda_i,\mu_i) + \frac{1}{2}\frac{\partial \varphi_{i-1}}{d\mu_i}(\mu_{i-1},\mu_i) + \frac{1}{2}\frac{\partial \varphi_i}{d\mu_i}(\mu_i,\mu_{i+1}). \tag{S6}$$

Here, $\gamma_T = 10\text{Pa}\cdot\text{s}$ is the transverse friction coefficient. Note that either the third or fourth term is omitted for the half-sarcomeres at both ends. From the longitudinal mechanical equilibriums at the interfaces of the half-sarcomeres and at the right end and from the transversal mechanical equilibrium in each half-sarcomere, the following equations must be fulfilled:

$$\begin{cases} T_{L,i} - T_{L,i+1} = 0, \quad i = 1,\cdots,ns-1 \\ k_L(L_{ns} - \bar{L}) - T_{L,ns} = 0 \\ \gamma_T \dot{\mu}_i + T_{T,i} = 0, \quad i = 1,\cdots,ns \end{cases}, \tag{S7}$$

where ns is the total number of half-sarcomeres.

The variables in Equation (S7) consist of the positions of half-sarcomere boundaries $\boldsymbol{L} =$

$(L_0, L_1, \cdots, L_{ns})^T$ and transverse stretches $\boldsymbol{\mu} = (\mu_1, \cdots, \mu_{ns})^T$. The longitudinal stretches are given by

$$\lambda_i = 2\frac{L_i - L_{i-1}}{SL_0}, i = 1, \cdots, ns, \quad \text{(S8)}$$

where the sarcomere length $SL_0 = 1.9\mu\text{m}$ in the unloaded condition. We assumed that the left end (i = 0) was fixed ($L_0 \equiv 0$) and that the right end ($i = ns$) was connected to a spring with the spring constant k_L. $\bar{L}$ is the length of the myofibril at which the spring force is zero. For the myofibril model consisting of 40 half-sarcomeres, $k_L = 30$ kPa/μm and $\bar{L} = 1.07 \cdot ns \cdot SL_0/2$ were adopted. These parameters were adjusted to reproduce the experimental data. For the numerical simulation of the single half-sarcomere model, $k_L = 30$ kPa/μm and $\bar{L} = 2.2 \cdot SL_0/2$ were adopted.

Active Contractile Tension

All the parameter values adopted in the actomyosin complex model described here are listed in Table S1. To determine the active contractile tension T_{act}, we applied a stochastic crossbridge cycling model [Washio2019] consisting of the five states (detachment, weak binding, prestroke, poststroke, and rigor states) depicted in Fig. 4. First, we describe the transitions between the strong binding states (the prestroke, poststroke, and rigor states), which are related to active tension. In this model, the rate constants of the power stroke ($k_{+\alpha}, \alpha = 3,4$) and the stroke reversal ($k_{-\alpha}, \alpha = 3,4$) are determined as a function of the rod strain such that they fulfill the relationship given by the following statistical equilibrium:

$$\frac{k_{+\alpha}(x)}{k_{-\alpha}(x + s_\alpha)} = \exp\left(\frac{E_\alpha + W_{rod}(x) - E_{\alpha+1} - W_{rod}(x + s_\alpha)}{k_B T}\right), \quad \text{(S9)}$$

where k_B and T denote the Boltzmann constant and the temperature, respectively, and E_α and $E_{\alpha+1}$ are the free energies of the actomyosin complex before and after the power stroke, respectively. The stroke distance s_α. x is the rod strain before the power stroke. W_{rod} is the strain energy in the myosin rod. In our numerical model, we put n_F one-dimensional filament pairs in each half-sarcomere to determine the active tension at time t generated by the bound myosin molecules, as follows:

$$ {}^{t}T_{\text{act}} = \frac{2}{SA_0 \cdot n_F} \sum_{b=1}^{n_F} \sum_{a=1}^{n_M} {}^{t}\delta_{A,a,b} \frac{dW_{rod}}{dx} \left({}^{t}x_{a,b} \right). \quad \text{(S10)} $$

Here, ${}^{t}\delta_{A,a,b} = 1$ if the myosin head is in the attached states; otherwise, ${}^{t}\delta_{A,a,b} = 0$. n_M is the number of myosin molecules arranged on a thin filament at regular intervals. SA_0 is the cross-sectional area per thin filament in an unloaded half-sarcomere. The factor of two comes from the fact that our one-dimensional model corresponds to one of the double spirals of actin monomers along the thin filament [Washio2016]. The number n_M is determined under this assumption.

As the forms of feedback from the sarcomeric dynamics to the actomyosin dynamics, the power stroke and power stroke reversal are affected by the half-sarcomere length change through the rod strain. The relation between the time transients of the molecular and sarcomeric variables is expressed by

$$ {}^{t}x = {}^{t_A}x + {}^{t}s - \int_{t_A}^{t} {}^{u}\dot{z}\, du. \quad \text{(S11)} $$

Here, t_A is the most recent time at which the myosin molecule was attached, ${}^{t_A}x$ is the initial strain at the attachment, ${}^{t}s$ is the total power stroke distance after the attachment, and ${}^{u}\dot{z}$ is the half-sarcomere shortening velocity given by

$$ {}^{u}\dot{z} = -\frac{SL_0}{2} {}^{u}\dot{\lambda}, \qquad t_A \leq u \leq t. \quad \text{(S12)} $$

Thus, half-sarcomere shortening ($\dot{z} > 0$) implies a decrease in rod strain resulting in facilitation of the power stroke transition (increase in $k_{+\alpha}(x)/k_{-\alpha}(x + s_\alpha)$), while half-sarcomere lengthening ($\dot{z} < 0$) implies an increase in rod strain, resulting in facilitation of stroke reversal (decrease in $k_{+\alpha}(x)/k_{-\alpha}(x + s_\alpha)$).

In this study, the rate constants for the power stroke and stroke reversal were determined based on the following temporary rates $\hat{k}_{+\alpha}$ and $\hat{k}_{-\alpha}$:

$$ \hat{k}_{+\alpha}(x) = h_\alpha \exp\left(\frac{E_\alpha + W_{rod}(x) - E_{\alpha+1} - W_{rod}(x + s_\alpha/2)}{k_B T} \right), \alpha = 3,4 \quad \text{(S13)} $$

$$ \hat{k}_{-\alpha}(x + s_\alpha) = h_\alpha \exp\left(\frac{W_{rod}(x + s_\alpha) - W_{rod}(x + s_\alpha/2)}{k_B T} \right), \alpha = 3,4 \quad \text{(S14)} $$

Here, E_α and $E_{\alpha+1}$ are the free energies in the myosin head before and after the power stroke transition, respectively. $W_{rod}(x + s_\alpha/2)$ is introduced to take the energy barrier between the two states before and after the power stroke into account [Washio2017]. Note that the contribution of the free energy in the myosin head at the barrier between the states before and after the power stroke is included in the constant h_α. The power stroke transitions are thought to accompany the release of Pi and ADP from the nucleotide binding pocket in the myosin head at the first and second power strokes, respectively [Wulf2016]. If that is case, the reversal stroke transitions must accompany the re-binding of these molecules. Therefore, it is reasonable to suppose that the transition rates are limited by the rates of these chemical reactions. We assumed the upper bounds due to these limitations from the release and the re-binding, which are given by $\bar{k}_{+\alpha}$ and $\bar{k}_{-\alpha}$, respectively. With these upper limits, the temporary rate constants given above are modified as follows:

$$k_{+\alpha}(x) = \begin{cases} \bar{k}_{+\alpha}, & x \leq \bar{x}_{+\alpha} \\ \hat{k}_{+\alpha}(x), & \bar{x}_{+\alpha} < x \leq \bar{x}_{-\alpha} \\ \dfrac{\hat{k}_{+\alpha}(x)\bar{k}_{-\alpha}}{\hat{k}_{-\alpha}(x+s_\alpha)}, & x > \bar{x}_{-\alpha} \end{cases} \quad \text{(S15)}$$

$$k_{-\alpha}(x + s_\alpha) = \begin{cases} \dfrac{\hat{k}_{-\alpha}(x+s_k)\bar{k}_{+\alpha}}{\hat{k}_{+\alpha}(x)}, & x \leq \bar{x}_{+\alpha} \\ \hat{k}_{-\alpha}(x+s_\alpha), & \bar{x}_{+\alpha} < x \leq \bar{x}_{-\alpha} \\ \bar{k}_{-\alpha}, & x > \bar{x}_{-\alpha} \end{cases}. \quad \text{(S16)}$$

Here, $\bar{x}_{+\alpha}$ and $\bar{x}_{-\alpha}$ are the strains at which the temporary rates reach the upper limits ($\hat{k}_{+\alpha}(\bar{x}_{+\alpha}) = \bar{k}_{+\alpha}, \hat{k}_{-\alpha}(\bar{x}_{-\alpha} + s_\alpha) = \bar{k}_{-\alpha}$).

The elastic force of a myosin rod is nonlinear with respect to the strain, as described by Kaya et al. [Kaya2010]. We assumed that a myosin rod behaves as a linear spring for positive stretches, whereas nonlinear behavior is introduced for negative stretches because of the slack region along the myosin rod. The strain energy W_{rod} is given by integrating the force F_{rod} from $x = 0$, defined by

$$F_{rod}(x) = \begin{cases} b_{xb}k_{xb}(x+\xi_1) - F_1, & x < -\xi_1 \\ \dfrac{k_{xb}}{a_{xb}}(\exp(a_{xb}x) - 1), & -\xi_1 \leq x < 0, \\ k_{xb}x, & x \geq 0 \end{cases} \quad \text{(S17)}$$

where a_{xb} and F_1 are determined from the other parameters so that the function F_{rod} and its

first derivative are continuous at $\xi = 0$ and $-\xi_1$:

$$\begin{cases} a_{xb} = -\dfrac{(\ln b_{xb})}{\xi_1} \\ F_1 = \dfrac{k_{xb}(1 - \exp(-a_{xb}\xi_1))}{a_{xb}} \end{cases} \quad \text{(S18)}$$

In our model, we assumed that attachment (the transition from the weak binding state to the prestroke state in Fig. 4c) is allowed only in the single overlapping region of the thin and thick filaments. We also assumed that myosin molecules are arranged on a thick filament at regular intervals except in the bare zone (B-zone). Therefore, the myosin head ($\#a$) is situated in the single overlapping region if and only if the following condition is fulfilled:

$$\max(LA - HSL, HSL - LA) \leq \frac{LB}{2} + \frac{2(a - 0.5)}{n_M(LM - LB)} \leq HSL. \quad \text{(S19)}$$

Here, the middle term is the distance from the center of the sarcomere; LM, LB and LA are the lengths of the thick filament, the B-zone and the thin filament, respectively; and $HSL = (\lambda \cdot SL_0/2)$ is the half-sarcomere length for stretch λ. The parameters for the sarcomere geometry were determined for a cardiac sarcomere [Kolb2016, Lodish, Rice2008, Rodriguez1993].

The thin filament is divided into n_T segments called troponin/tropomyosin (T/T) units (Fig. 1b). Three states, called Ca-off, Ca-on*, and Ca-on, are assumed by each T/T unit. The transitions between the states of the T/T unit are determined by $[\mathrm{Ca}^{2+}]$ and the four parameters K_{on}^*, K_{on}, K_{off}^* and K_{off}, as shown in Fig. 4d. The transitions between the detachment and weak binding states (Fig. 4c) are affected by the status of the T/T unit above it through modifications of K_{np} and K_{pn} as well as by the state of the neighboring myosin heads through the integer ng, where ng $(= 0, 1, \text{or } 2)$ is the number of neighboring myosin heads in the weak binding or the three strong binding states. The corresponding T/T unit index τ of the a-th myosin head is given by:

$$\tau = \mathrm{int}\left(\frac{z + 0.5LB + (a - 0.5)S_M + -(0.5SL_0 - LA)}{S_T}\right). \quad \text{(S20)}$$

Here, $S_M = 0.5(LM - LB)/n_M$ is the spacing of the myosin heads, and $S_T = LA/n_T$ is the spacing of the T/T units. Note that the corresponding T/T unit exists only if $1 \leq \tau \leq n_T$. Under the above correspondence, the factors K_{np} and K_{pn} of the rate constants are given by:

$$K_{\mathrm{np}} = \begin{cases} \delta_{OV} K_{\mathrm{np1}} & \text{if the T/T unit above is in the Ca-on state,} \\ \delta_{OV} K_{\mathrm{np0}} & \text{otherwise.} \end{cases} \quad \text{(S21)}$$

$$K_{\mathrm{pn}} = \begin{cases} K_{\mathrm{pn1}} & \text{if the T/T unit above is in the Ca-on state,} \\ K_{\mathrm{pn0}} & \text{otherwise.} \end{cases} \quad \text{(S22)}$$

Here, $\delta_{OV} = 1$ if the myosin head is located at the single overlapping region with the thin filament; otherwise, $\delta_{OV} = 0$. With the factors γ^{ng} and γ^{-ng} $(\gamma = 60)$, this represents the nearest-neighbor cooperativity of the myosin heads, following the methods of Rice (2003), which plays an important role in the force-pCa relationship [Washio2018]. We assumed that one thin filament in the three-dimensional arrangement corresponds to two thin filaments in our half-sarcomere model. This is because we assumed that cooperative behavior exists along the tropomyosin and tropomyosin molecules wrapped around the thin filament in a double spiral fashion, and only one of the spirals is considered in our half-sarcomere model. The constants K_{np0}, K_{np1}, K_{pn0}, and K_{pn1} are determined from Q, K_{basic}, and μ, as follows:

$$\begin{cases} K_{\mathrm{np0}} = F_K(SL) \dfrac{Q K_{\mathrm{basic}}}{\mu}, \\ K_{\mathrm{np1}} = F_K(SL) Q K_{\mathrm{basic}}, \\ K_{\mathrm{pn0}} = K_{\mathrm{pn1}} = K_{\mathrm{basic}} \gamma^2. \end{cases} \quad \text{(S23)}$$

Here, $\mu > 1$ controls the degree of crossbridge inhibition for T/T units in states other than Ca-on, and Q controls the ratio of binding states of the myosin heads. The greater the value of Q, the larger the ratio of binding states for a given [Ca^{2+}]. In this study, we assumed that elevated temperature facilitates the transition from the detachment state to the weak binding state by relaxing the binding inhibition of tropomyosin. This effect was introduced by multiplying the temperature-dependent coefficient c_T to define Q.

$$Q = c_T Q_0 \quad \text{(S24)}$$

$c_T = 1$ was adopted for the case before heating, while $c_T = 2$ was adopted for the case after heating. To reproduce the sarcomere length ($SL = SL_0 \lambda$) dependence in the active contraction tension, the following function $F_K(SL)$ was multiplied to define K_{np0} and K_{np1}:

$$F_K(SL) = \begin{cases} 1 & , SL \geq SL_Q, \\ 1 - \alpha_Q (SL_Q - SL), & SL < SL_Q. \end{cases} \quad \text{(S25)}$$

α_Q=1[1/μm] and SL_Q=2.2μm were adopted in this study.

The rate constants of attachment (k_{+2}) and detachment (k_{-2}) are given based on the assumed free energies E_2 and E_3, respectively, in the weak binding state and the prestroke state, as follows:

$$k_{+2} = k_{-2}\exp\left(-\frac{E_2 - E_3}{k_B T}\right). \quad \text{(S26)}$$

The initial rod strain at the attachment is given stochastically from the Boltzmann distribution determined by the rod strain energy $W_{rod}(x)$ [Washio2016]. The detachment rate constant from the rigor state to the detachment state is assumed to be strain-dependent for the negative strain, as follows:

$$k_{+5}(x) = \begin{cases} d_{rig0}\exp(-F_{rod}(x)/F_d), & x < 0 \\ d_{rig0}, & x \geq 0 \end{cases}. \quad \text{(S27)}$$

F_d=6pN was applied in this study.

We also took the forced detachment due to the extreme strain of the myosin rod into account with the rate constant:

$$d_{for}(x) = \begin{cases} 0, & x < x_{for} \\ c_{for}\left(\exp\left(a_{for}(x - x_{for})\right) - 1\right), & x \geq x_{for} \end{cases}, \quad \text{(S28)}$$

where a_{for}=0.3 [1/nm] and x_{for}=10 nm. c_{for} is listed in Table 1.

Here, we assumed that the forced detachment from the prestroke state is directed to the weak binding state, while the forced detachment from the poststroke or rigor state is directed to the detachment state. For detachment from the prestroke state and the rigor state, the rate constant in Equation (S28) was added to the original rate constant.

References

[Ertbjerg2017] Ertbjerg, P. & Puolanne, E., Muscle structure, sarcomere length and influences on meat quality: A review, Meat Sci. **132**, 139–152 (2017).

[Kaya2010] Kaya, M., & Higuchi, H.. Non-linear elasticity and an 8 nm working stroke of single myosin molecules in myofilaments, Science **329**(5992), 686–689 (2010).

[Kolb2016] Kolb, J., Li, F., Methawasin, M., Adler, M., Escobar, Y. N., Nedrud, J., Pappas, C. T., Harris, S. P. & Granzier, H., Thin filament length in the cardiac sarcomere varies with sarcomere

length but is independent of titin and nebulin, J Mol Cell Cardiol. **97**, 286–294 (2016).
[Konhilas2002] Konhilas, J. P., Irving, T. C., & de Tombe, P. P., Length-dependent activation in three striated muscle types of the rat, J Physiol. **544**(1),225–236 (2002).
[Lodish2000] Lodish, H., Berk, A., Zipursky, S. L., Matsudaira, P., Baltimore, D. & Darnell, J., Molecular Cell Biology, 4th edition, New York: W. H. Freeman (2000).
[Rice2008] Rice J. J., Wang, F., Bers, D. M. & de Tombe, P. P., Approximate model of cooperative activation and crossbridge cycling in cardiac muscle using ordinary differential equations, Biophys J. **95**(5), 2368–2390 (2008).
[Rodriguez1993] Rodriguez, E. K., Omens, J. H., Waldman, L. K. & McCulloch, A. D., Effect of residual stress on transmural sarcomere length distributions in rat left ventricle, Am J Physiol. **264**(4 Pt 2), H1048–1056 (1993).
[Sato2013] Sato, K., Kuramoto, Y., Ohtaki, M., Shimamoto, Y. & Ishiwata, S., Locally and globally coupled oscillators in muscle, Phys Rev Lett. **111**(10), 108104 (2013).
[Telley2006] Telley, I. A., Denoth, J., Stüssi, E., Pfitzer, G. & Stehle, R., Half-Sarcomere dynamics in myofibrils during activation and relaxation studied by tracking fluorescent markers, Biophys J. **90**(2), 514–530 (2006).
[Washio2016] Washio, T., Yoneda, K., Okada, J., Kariya, T., Sugiura, S. & Hisada, T., Ventricular fiber optimization utilizing the branching structure, Int. J. Numer. Method Biomed. Eng. **32**:e02753 (2016).
[Washio2017] Washio, T., Hisada, T., Shintani, S. A., & Higuchi, H., Analysis of spontaneous oscillations for a three-state power-stroke model, Phys Rev E. **95**(2-1), 022411 (2017).
[Washio2018] Washio, T., Sugiura, S., Kanada, R., Okada, J. & Hisada, T., Coupling Langevin dynamics with continuum mechanics: Exposing the role of sarcomere stretch activation mechanisms to cardiac function, Front. Physiol. **9**, 333 (2018).
[Washio2019] Washio, T., Shintani, S. A., Higuchi, H., Sugiura, S. & Hisada, T. Effect of myofibril passive elastic properties on the mechanical communication between motor proteins on adjacent sarcomeres. *Scientific reports*. **9**, 9355 (2019).
[Wulf2016] Wulf, S. F., Ropars, V., Fujita-Becker, S., Oster, M., Hofhaus, G., Trabuco, L. G., Pylypenko, O., Sweeney, H. L., Houdusse, A. M. & Schröder, R. R., Force-producing ADP state of myosin bound to actin, Proc Natl Acad Sci U S A. **113**(13), E1844–E1852 (2016).

Table S1 Parameters for actomyosin dynamics. "Adjusted" in the Reference column means that the values were adjusted to reproduce the phenomena. For the factor R_0, the difference values 4 and 7 were applied for the normal temperature and for the HSO, respectively.

Parameter	Value	Unit	Reference	Parameter	Value	Unit	Reference
**ATP hydrolysis energy**				**Sarcomere geometry**			
E_{ATP}	76.5	$\mathrm{pN \cdot nm}$	[Washio2016]	SL_0	1.9	μm	[Rodriguez1993]
k_B	0.0138	$\mathrm{pN \cdot nm/°C}$		LM	1.65	μm	[Rice2008]
**Stroke sizes and free energies**				LB	0.16	μm	[Lodish2000]
$s_3,\ s_4$	5.5	nm	[Washio2016]	LA	1.0	μm	[Kolb2016]
E_{Pre}	E_{ATP}	pN/nm	[Washio2016]	SA_0	693	nm^2	[Sato2013]
E_3	$1.05E_{\mathrm{Pre}}$	pN/nm	[Washio2016]	**Force regulation through T/T unit**			
E_4	$0.82E_{\mathrm{ATP}}$	pN/nm	[Washio2016]	K_{on}^*	$50R_0$	1/s	adjusted
E_5	0	pN/nm	[Washio2016]	K_{off}^*	$5R_0$	1/s	adjusted
**Rod strain energy W_{rod}**				K_{on}	$90R_0$	1/s	adjusted
k_{xb}	2.0	pN/nm	[Washio2016]	K_{off}	$60R_0$	1/s	adjusted
b_{xb}	0.05	unitless	[Washio2016]	K_{basic}	20	1/s	[Washio2016]
ξ_1	4.35	nm	[Washio2016]	Q_0	2	unitless	[Washio2016]
**Power-stroke transitions**				μ	20	unitless	[Washio2016]
h_3	$20R_0$	1/s	adjusted	γ	60	unitless	[Washio2018]
h_4	$0.1R_0$	1/s	adjusted	**Sarcomere friction**			
$\bar{k}_{+3}, \bar{k}_{+4}$	$5000R_0$	1/s	adjusted	γ_L	0.01	kPa·s	[Washio2017]
$\bar{k}_{-3}, \bar{k}_{-4}$	$80R_0$	1/s	adjusted	γ_T	0.01	kPa·s	adjusted
**Detachment transitions**				**Number of elements in a sarcomere**			
k_{-2}	$2000R_0$	1/s	[Washio2016]	n_M	38	unitless	[Washio2016]
$d_{rig,0}$	$50R_0$	1/s	[Washio2016]	n_T	32	unitless	[Washio2016]
c_{for}	R_0	1/s	adjusted	n_F	4096	unitless	adjusted
